# 

**Published:** 1822-01

**Authors:** 


### THE LONDON MEDICAL AND PHYSICAL JOURNAL.

CONTAINING

ORIGINAL CORRESPONDENCE OF EMINENT PRACTITIONERS,

AND

CRITICAL ANALYSIS OF NEW WORKS

RELATING TO MEDICINE, SURGERY, MIDWIFERY, CHEMISTRY, PHARMACY, BOTANY, AND NATURAL HISTORY.

---

Successively conducted by

T. BRADLEY, M.D.
A. F. M. WILLICH, M.D.
R. BATTY, M.D.
A. A. NOEHDEN, M.D.
DRS. ARNEMAN AND PFAFF,
J. ADAMS, M.D.
W. SHEARMAN, M.D.
W. ROYSTON, ESQ.
S. FOTHERGILL, M.D.
W. HUTCHINSON, M.D.

AND NOW EDITED BY

A. B. GRANVILLE, M.D. F.R.S. F.L.S. M.R.I.

Physician in Ordinary to his Royal Highness the Duke of Clarence; Licentiate of the Royal College of Physicians; Principal Physician to the Royal Infirmary for Children; Physician-Accoucheur to the Westminster General Dispensary, and to the Benevolent Institution; Honorary Member of the Royal Academy of Medicine of Madrid; Foreign Associate of the Royal Academy of Sciences of Naples; Member of the Philosophical and Literary Society of Manchester; of the Geological Society of London; of the Philomatic and Philotechnic Societies of Paris; Member of the Society of the Faculty of Medicine of Paris, of the Societé Medicale d'Emulation, and the Cercle Medical of the same town; Correspondent of the Prussian Physical Society of the Lower Rhine; Member of the Imperial Academy of Lucca; of the Societies of Florence, Marseilles, Leghorn, Pistoja, Padua, Venice, &c. &c.

---

VOL. XLVII.

FROM JANUARY TO JUNE, 1822.

Et quoniam variant morbi, variabimus artes;
Mille mali species, mille salutis erunt.

SURGEON GENERAL'S OFFICE LIBRARY
4629

*LONDON:*

PRINTED FOR THE PROPRIETORS,

*By J. and C. Adlard, 23, Bartholomew Close;*

PUBLISHED BY J. SOUTER, 73, ST. PAUL'S CHURCH-YARD;

AND MAY BE HAD OF ALL BOOKSELLERS.

W1
LO31

### ANALYTICAL TABLE OF THE CONTENTS

OF

THE FORTY-SEVENTH VOLUME

OF THE

**London Medical and Physical Journal.**

### DESCRIPTIVE ANATOMY.

| | PAGE |
|---|---|
| Case of Transposition of the Abdominal Viscera | 2 |
| Abdominal and Thoracic Viscera | 3 |
| Malformation in a Fetus | ib. |
| Comparative Anatomy of the Brain | ib. |
| Anatomical Theory of Monstrosities | ib. |
| Works on Anatomy, by Hippolyta and Jules Cloquet | ib. |
| Addition to Bichat's Anatomy | 77 |
| Systéme Anatomique, by Cloquet | 4 |
| On the Organ of Hearing | 4, 438 |
| Manual of Anatomy | 4 |
| On the Organization of the Heart | 5 |
| On a Man having two Noses | ib. |
| On the Fascia Iliaca | 6 |
| Description of Parts concerned in the Operation of Lithotomy | ib. |
| On the Animal Tissues | ib. |
| On the Structure of the Human Eye, and use of the Marsupium in Birds | ib. |
| Of Aberrations in the Distribution of the Blood-vessels | ib. |
| Anatomico-Physiological Observations on the Epidermis | 7 |
| On the Urinary Organs of the Rana | 8 |
| On the Nerves going to the Mustachios of the Phoca | ib. |
| On the Peculiarities that distinguish the Manata from the Dugong of the West from that of the East Indies | ib. |
| On the Membrano-gelatinous Structure in the Acipenser | ib. |
| Account of a Girl born with six Fingers on each Hand and fifteen Toes | 16 |
| Remarkable Deviations in the Blood-vessels | 127 |
| On the Structure of the Chrystalline Lens | 346 |
| Anatomy and Physiology of the Facial Nerves | 432 |

### PHYSIOLOGY.

| | |
|---|---|
| On the Laws of Animal Life | 8, 9, 11, 12 |
| On the Mechanism of the Movements and Attitudes of Man | 9 |
| On the Rete Mucosum of the Negro | ib. |
| On the Physiology of Generation | ib. |
| Memoir on Vomiting | ib. |
| On the Transmission of Sounds to the Organ of Hearing by Facial Nerves | |
| On Sympathy | 11 |
| On Absorption by the Skin | ib. |
| On the Secretion of Urine | 12 |
| On the Strength of the Muscles | ib. |
| On the Blood and its Colouring Principle | ib. |
| On the Relation between the Form and Organization of the Portio Mollis of the seventh Pair and its Functions | ib. |

PAGE
On the Coagulation of the Blood . . . . . . . 12
On the Decomposition of Aliment . . . . . 184
Heat produced in the Skin by Chlorine . . . . . 349
On the Division of the Pneumo-gastric Nerves . . . . 391
Inquiry into the supposed Faculty of the Membranes of the Ovum to dilate the Os Uteri during Labour . . . . . . . 432
On Phrenology . . . . . . . . 469
Experiments on Absorption . . . . . . 471
On the Physiology of the Nervous System . . . . . 483

### PATHOLOGY.

OBSERVATIONS RELATIVE TO

SPINA BIFIDA . . . . . . . . 106

PARALYSIS of the Kidneys . . . . . . 107

YELLOW FEVER, state of the Skin . . . . . 113

OSTEO SARCOMA . . . . . . . . 213

VARICELLA and VARIOLA, identity of . . . . . 269

SPINAL IRRITATION . . . . . . . 301

MADNESS in a man, from inoculation of the Glanders . . 435

### PATHOLOGICAL ANATOMY.

Description of the Morbid Preparations in the Museum of the University of Padova . . . . . . . . . . 6

Natural Phimosis in an Infant . . . . . . . 7

On Monstrosities of the Spleen . . . . . . . ib.

On Hypertrophia of the Liver . . . . . . . ib.

On Carnification of the Gall-bladder . . . . . . ib.

Distension of the Œsophagus . . . . . . . ib.

Ossification of the Ventricular Aorta . . . . . . ib.

Description of an external Serous Cyst . . . . . . ib.

Case of Spina Bifida . . . . . . . . . 8

Sanguineous Apoplexy in a Child . . . . . . . 182

Internal Abdominal Hemorrhage . . . . . . . 261

Introsuscepted Cœcum . . . . . . . . 272

Tumor in the Cerebellum of a Child . . . . . . 273

Introsusception . . . . . . . . . . 274

Phthisis . . . . . . . . . . . 340

Hydrocephalus . . . . . . . . . . ib.

Marasmus . . . . . . . . . . . 341

Purpura . . . . . . . . . . . ib.

Schirrus Liver . . . . . . . . . . 342

Sudden Death from Rupture of the Heart . . . . . . 433

### NOSOLOGY.

Systematis Nosologici Novi exemplum Parvulum . . . . . 266

### SEMIOLOGY.

Dr. HALL's Work on Semiology . . . . . . . 401
Dr. DOUBLE's Work on ditto . . . . . . . 536

### HYGIENE.

On the Physical Education of Children . . . . . . . 27
Relative to the best Method of Warming and Ventilating Houses . . 28
the Population of Paris . . . . . . . 29
Medical Gymnastics . . . . . . . . ib.

PAGE

Relative to an attempt to inoculate the Plague . . . . . 29
Report of the National Vaccine Establishment . . . . 207
Strong Example of the protecting Power of the Vaccine against Small-pox 432

### THERAPEUTICS.

On Maximum Doses in Medicine . . . . . . . . 89
Prevailing Diseases . . . . . . . . . 338
OBSERVATIONS RELATING TO
ANASARCA, on Scarifications in . . . . . . 361
APOPLEXY . . . . . . . . 181
, supposed . . . . . . 367
ARACHNITIS . . . . . . . 495
ARTERITIS . . . . . . 18, 19
CALCULI . . . . . . . . . 520
CANCER . . . . . . . . . 155
UTERI . . . . . . . . 255
CEREBRAL AFFECTIONS, Articular Rheumatism, Epigastralgia, Submammary Pain with Dyspnœa, Cephalalgia, and foul Ulcer, cured by Ustion . . . . . . . 21
CHEST, Diseases of . . . . . . . 140
CHILDREN, Diseases of . . . . . 27, 77
CUTANEOUS DISEASES . . . . . 392, 482
DIABETES . . . . . . . . . 460
DYSPHAGIA . . . . . . . . 430
EAR, Diseases of . . . . . . . . 247
ERYSIPELAS . . . . . . . . 18
, on the application of Ointments to . . . 171
FEMALE DISEASES . . . . . . . 52
GANGRENE, HOSPITAL . . . . . . . 21
GASTRITIS . . . . . . . . . 19
GASTRO ENTERITIS . . . . . . . ib.
GOUT, on colchicum in . . . . . . 95, 459
HYDROCEPHALIC AFFECTIONS . . . . 18, 179, 183
HYDROPHOBIA, Preservatives against . . . . 84, 256
from a rabid Badger . . . . . 438
IMPETIGO, on Prussic Acid in . . . . . . 117
INFLAMMATION . . . . . . . . 518
INFLAMMATORY DISEASES . . . . . 22, 151
INFLUENZA . . . . . . . . . 158
INHALATIONS . . . . . . . . . 522
INTERMITTENT FEVER, on Prussiate of Iron in . . . 346
on Subnitrate of Bismuth in . . . 436
on Black Pepper in . . . . 431
IRRITATIONS . . . . . . 414
JAUNDICE, on Phosphoric Acid in . . . . . . 350
LEUCORRHŒA, on Cubebs in . . . . . . 434
LIVER, Diseases of . . . . . . 42, 123
MAL D'ESTOMAC . . . . . . . . 450
MELANCHOLIA . . . . . . . . . 298
NERVES, local Affection of . . . . . . 22
Diseases of the Nervous System . . . . 129
PALPITATION . . . . . . . . . 70
PARALYSIS cured by Lightning . . . . . . 523
PERICARDITIS . . . . . . . . 18
PHLEGMASIA . . . . . . . . . 514
DOLENS . . . . . . . 78
POISONS, NARCOTIC, Treatment of . . . . . 288
POISONING from Tincture of Cantharides . . . . 437
POLYPI, MUCOUS, on the external use of Tinct. Opii in . . 256
PORRIGO . . . . . . . . . 27, 146
PUERPERAL FEVER . . . . . . . . 517
SCARLATINA, Belladonna a preservative against . . 334

PAGE

STOMACH, Ulceration of . . . . . . . 19

and BOWELS diseased . . . . . . 246

STRICTURE of the RECTUM . . . . . 324

TÆNIA, use of Mare's Milk in . . . . . 523

TETANUS, successfully cured . . . . . 256

TIC DOULOUREUX, cured by Sulphate of Quinine . . 432

TROPICAL DISEASES . . . . . . 406

TYPHUS . . . . . . . . . 515

WHOOPING COUGH . . . . . . . 27

### CHIRURGERY.

Case of Laceration of the Colon . . . . . . 21
On Diseases of the Eyes . . . . . . . 28
Ears . . . . . . . 23
On Milk Abscess . . . . . . . . . 26
Amputation of the lower Jaw at its Articulation . . . . 260
Observations on Amputations . . . . . . 510
*Respecting the Operations for*
ARTIFICIAL ANUS . . . . . . . 109
EXTRACTING CALCULUS . . . . . 19, 20, 300
FOOT, partial Amputation of . . . . . . 20
BRONCHOCELE, by Seton . . . . . . ib.
CAROTID ANEURISM . . . . . . . ib.
GASTROTOMY . . . . . . . . 167
KNEE, foreign Body in . . . . . . . 22
REMOVAL of NECROSED BONE . . . . . . 21
OVARIAN SAC extirpated . . . . . . 281
SUTURES in contused Wounds . . . . . . 447
TRACHEOTOMY . . . . . . . 21, 433
THIGH, Fractures of the . . . . . . 441
TUMOR . . . . . . . . . 21
in the Rectum . . . . . . . . 7
VARICOSE VEINS . . . . . . . . 161
CÆSARIAN OPERATION, successful . . . . . 25
New Instruments for Nasal Polypi . . . . . . . 84
Cataract . . . . . . . 168
Excision of the Neck of the Womb . . . 255
Instruments discovered in Pompeii . . . . . . 353

### OBSTETRICISM.

Obstetrical Researches, Treatises on Midwifery . . . . 23
Case of Ruptured Vagina during Parturition . . . . 24
Loss of the Os Uteri during Labour . . . . ib.
On the spontaneous Evolution of the Fetus . . . . 25
Case of a Woman who died undelivered in consequence of a Rupture of the Uterus . . . . . . . . . ib.
Placenta expelled before the Child . . . . . ib.
Cæsarean Operation, successful . . . . . . ib.
Impediment to Delivery, from the Umbilical Cord round the Neck of the Fetus . . . . . . . . . ib.
Impediment to Delivery, from the spasmodic Contraction of the Neck of the Womb round the Fetus . . . . 25, 26
Posthumous Parturition . . . . . . . 26
On Utero-vaginal Diseases . . . . . . . . 27
Fatal cases of difficult Parturition from locking of the Heads of Twins . 210
from narrow Pelvis and Contraction of the Uterus . . . 277
from Laceration of the Cervix Uteri . . . . . 373
Report of the Practice of Midwifery at the Westminster Dispensary 282, 374
On Encysted Tumors in the Ovarium, discharged through the Abdomen after Suppuration . . . . . . . . 463

### MEDICAL JURISPRUDENCE AND MEDICAL POLICE.

PAGE

Treatise on Juridical Medicine . . . . . . . . 30

Case of Poisoning by Arsenic, successfully treated . . . ib.

Fatal case of Poisoning by Bleaching Liquid . . . . ib.

On the Detection of Frauds practised by Venders of Milk . . 294

Table of Suicides in Paris . . . . . . . . 128

Tests for Arsenic . . . . . . . . . 260

Statistical Inquiries respecting Paris . . . . 66, 128

Rapports et Consultations de Medecine Legale . . . . 75

### CHEMISTRY.

Analysis of the Roots of Black Hellebore . . . . . . 47

Alvine Concretions . . . . . . . 82

a new Mineral called Thompsonite . . . . 169

the Humour of Porrigo . . . . . . 350

Tea . . . . . . . . . ib.

Croton Oil . . . . . . . . 378

a Pigment from the Tomb of Psammis . . . 35

Improvement in Wolfe's Apparatus . . . . . . ib.

On a new Pyrometer . . . . . . . . ib.

Experiments on the chemical Changes of Fruits . . . . ib.

Dictionary of Chemistry . . . . . . . . ib.

Thousand Experiments on Chemistry . . . . . . ib.

Elements of Chemical Science . . . . . . . 36

On a new Combination of Chlorine and Carbon . . . . ib.

On Sulphuretted Azote . . . . . . . . 189

On Alloys of Steel with different Metals . . . . . 343

On Nitrogen in Mineral Waters . . . . . . . 345

Spontaneous Explosion of Chlorine and Hydrogen . . . . 349

On the Action of Sulphuret of Carbon on Phosphorus and Carbon . ib.

On a new Metal . . . . . . . . . . 434

Solution of Oxide of Copper in Ammonia . . . . . . 436

On a Deposit found in the Waters of Lucca . . . . ib.

Composition of Oxalic Acid . . . . . . . . 526

### MATERIA MEDICA, PHARMACY, MEDICAL BOTANY, AND SOME POINTS IN NATURAL HISTORY.

On the virtues of Hydrocyanic Acid . . . . . . 31
Colchicum . . . . . . . 32
Cubebs . . . . . . . . ib.
Iodine . . . . . . . . ib.
Oil of Croton . . . . . . . 33
Terebinthinous Remedies . . . . ib.
Pomegranate Bark in Tænia . . . . ib.
Swietenia Febrifuga . . . . . . ib.
Sulphate of Quinine . . . . . . ib.
Tar Fumigation . . . . . . . 34
Tincture of common Poppy . . . . ib.
Spinea Tormentosa of Linnæus . . . . . ib.
On Arsenic in the Preparations of Antimony used in Medicine . . ib.
On Cantharides and on Scarabæus Melalontha, as Preservatives against Hydrophobia . . . . . . . 84, 256
Specimen of the great-eared Owl . . . . . . . 85
Artificial Mineral Waters . . . . . . . . 201
On the Phosphorescence of the Lampyris Italica . . . . 261
Proposal for a new Classification of Medicines . . . . 289

| | PAGE |
|---|---|
| On Croton Oil | 378 |
| On the Preparation of Ethiop's Mineral | 435 |
| On Bitter-almond Emulsion as a Substitute for Hydrocyanic Acid | 438 |
| Cheltenham Waters | 156 |
| Botany of the United States | 236 |
| Preparations of Gold | 248 |
| On the use of the Nitrate of Silver in Medicine | 524 |
| Results obtained by the use of Iodine in the Clinico-medical School, Padua | ib. |
| Chameleons, or Lizards of Cashou | 527 |

### MISCELLANEA.

| | |
|---|---|
| On the Connexion of Electric and Magnetic Phenomena | 50, 85 |
| Account of Flatted Skulls | 83 |
| Instance of Longevity | 84 |
| Cholera Morbus at Samarung | ib. |
| Royal marks of Favour to Dr. BALLY | 85 |
| Report of the Liverpool Ophthalmic Infirmary | 86 |
| Eighth Report of the Clinical Institution at Halle | 159 |
| Pharmacopœia Hanoveriana | 160 |
| Infantile Polysarcia | 170 |
| Curious mode of calculating a Man's Life | ib. |
| Pearl found in the Human Body | 171 |
| Spermacetic Animalculi | ib. |
| Suicide produced solely by a Self-persuasion of its Hereditary Predisposition | 298 |
| Unparalleled instance of Suicide by Starvation | 205 |
| BROUSSAIS' new Medical Journal | 257 |
| Population of Great Britain | 256 |
| Lithographic Botany | 259 |
| Preservation of Milk | ib. |
| Animal Specimens | 436 |
| Effects of Lightning | 260 |
| Difference of Temperature in the same Room at different Altitudes | 261 |
| Curious Atmospheric Phenomena | ib. |
| A new Lactometer | 295 |
| First appearance of the Boa Constrictor in the Island of St. Vincent | 346 |
| Cretinism in Wurtemberg | ib. |
| Dark-brown Streak on the Sea, occasioned by Crabs | 349 |
| A Man struck Blind and Dumb by Lightning | 369 |
| On the Setting of Surgical Instruments | 435 |
| Prize Questions | 162, 163, 344, 521 |
| Academical Proceedings, National and Foreign | 80, 162, 254, 342, 432, 520 |
| Meteorological Journals | 87, 175, 263, 351, 439, 527 |
| Monthly Catalogues of Books | 87, 176, 264, 351, 440, 528 |
| Notices to Correspondents | 88, 176, 264, 352, 440, 528 |

### LIST OF PLATES.

| | |
|---|---|
| Mr. JUKES's Case of Spina Bifida | *to face page* 106 |
| Case of Introsusception | 274 |
| Dr. GIBSON's Apparatus for fractured Thigh | 441 |

### NAMES OF AUTHORS,

*Of whose Works, Observations, &c. either a detailed Account, or more or less general View, is given in*

### THE FORTY-SEVENTH VOLUME OF THE LONDON MEDICAL AND PHYSICAL JOURNAL.

PAGE

ALEXANDER, Mr. of *Dumferline*, case of difficult Parturition, from the Locking of the Heads of Twins . . . . . . 211
AMARD, Mr. of *Lyons*, Association Intellectuelle . . . . . 497
ANGLADA, M. on the Existence of Nitrogen in Mineral Waters . . . 345
ASPRAY, Mr. case of Laceration of the Neck of the Cervix Uteri . 372
AUTHENAC, M. Defence des Médecins Français, &c. . . . 76
BAMPFIELD, Mr. of *London*, on the Superiority of small over large Doses of Colchicum in Gout . . . . . . . . 95
Cases illustrating the Identity of Small-pox and Varicella . . 269
BARLOW, Mr. Essays on Surgery and Midwifery . . . . . 303
BARTON, Professor, of *Philadelphia*, Vegetable Materia Medica of the United States . . . . . . . . . 236
BECLARD, M. of *Paris*, Additions to Bichat's Anatomy . . . 77
and Dr. JULES CLOQUET, Anatomie de l'Homme . . 503
BELL, Mr. C. paper on the Nerves, read to the Royal Society . . 432
BERENDT, Dr. of *Austria*, on the use of Belladonna as a Preservative from Scarlet Fever . . . . . . . . . 336
BERZELIUS, Mr. of *Sweden*, on the Solution of Oxide of Copper in Ammonia 436
BIGELOW, Prof. of the *United States*, American Medical Botany . . 236
BLANE, Sir GILBERT, of *London*, Tribute of gratitude and esteem to . 172
BÖDTCHER, Dr. of *Denmark*, on the Employment of Camphor in several Diseases . . . . . . . . . . 250
BRANDE, Mr. of *London*, analysis of Tea . . . . . 350
BRESCHET, Dr. of *Paris*, Considerations sur les Melanoses . . 156
BRICHETEAU, Mr. of *Paris*, on an unsuccessful case of Tracheotomy . 433
BRODIE, Mr. of *London*, Introductory Lecture at the College of Surgeons 154
Experience of the effects of Ointments in Erysipelas . . 171
BROUSSAIS, Dr. of *Paris*, his Medical Journal announced . . 257
Histoire des Phlegmasies . . . . . . 514
BUCKLAND, Rev. Mr. paper on the Bones of Antediluvian Animals, read to the Royal Society . . . . . . . . . 254
BUSH, Mr. FRANCIS, Remarks on Mr. Yeatman's Reply . . . . 357
On the use of the Emulsion of Bitter Almonds, as containing Hydrocyanic Acid . . . . . . . . . 438
CADET DE GASSICOURT, of *Paris*, death of . . . . . 100
CALLUP, Dr. of *Philadelphia*, case of a large mass of an Amalgam of Tin and Mercury retained in the Bowels . . . . . . 161
CANELLA, Dr. of *Milan*, Cenni sull' Estirpatione della Bocca e del Collo dell' Utero, &c. . . . . . . . . . 253
CARTONI, of *Tuscany*, della Manièra piu Atta a Curare radicalmente le Varici, &c. . . . . . . . . . 161
CASPAR, Dr. of *Prussia*, Commentarius de Phlegmatia Dolente . . 78
CHISHOLM, Dr. on Tropical Diseases . . . . . . 406
CHILDREN, Mr. of *London*, analysis of an Alvine Concretion . . 82
CLARKE, Mr. C. of *London*, observations on the Treatment of Diseases attended by Discharges . . . . . . . . 52
CLOQUET, HYP. of *Paris*, Histoire des Animaux et de leur Produits, &c. . 520
CLOQUET, JULES, of *Paris*, sur les Calcules Urinaires . . . ib.
COSTERTON, Mr. of *Yarmouth*, case of Hydrocephalus cured by Pressure 185
CURTIS, Mr. of *London*, cases illustrative of the Treatment of Diseases of the Ear . . . . . . . . . . . 247
DAVY, Prof. of *Cork*, on an Instrument by which Frauds in the sale of Milk can be detected . . . . . . . . . 294

PAGE
Davy, Sir H. on a Deposit found in the Waters of Lucca . . 436
Desmoulins, Dr. of *Paris*, on the State of the Skin in Yellow Fever . 113
Recherches Anatomiques et Physiologique sur le Systeme Nerveux des Poissons . . . . . . . . 520
Doberainer, Mr. on the Composition of Oxalic Acid . . . 526
Dods, Dr. of *Worcester*, the Physician's Guide . . . . 71
Double, Dr. of *Paris*, Work on Semiology . . . . . 526
Dugdale, Mr. on Mr. Collier's proposed Method of restoring Patients poisoned by Narcotics . . . . . . . 288
Earle, Mr. of *London*, observations on Diseases of the Spine, read to the Royal Society . . . . . . . . 432
Falret, Mr. case of Melancholia . . . . . 298
Farr, Mr. of *London*, an Essay on the Fucus Helminthoides . . 155
Farraday and Stoddart, Messrs. memoir on the Alloys of Steel with Silver, Platinum, Palladium, Osmium, and Cromium . . 343
Fenuelle and Capron, analysis of Black Hellebore . . . 47
Filippi, Chevalier Joseph, a new Analytical Essay on Inflammation . 518
Fodera, M. on the Plagiarisms of Dr. Broussais, from Baglivi . . 76
Follickoffer, Dr. of *Baltimore*, Treatise on the Prussiate of Iron in Intermittent Fevers, announced . . . . . . 346
Forbes, Dr. of *Penzance*, Translation of Laennec's Work on Diseases of the Chest . . . . . . . . . . 140
Georget, Dr. of *Paris*, on the Physiology of the Nervous System . 485
Gibney, Dr. of *Cheltenham*, a Medical Guide to the Cheltenham Waters . 156
Gibson, Prof. of the *United States*, invention of an Instrument for Cataract 167
On Osteo-Sarcoma . . . . . . . . . 213
On an Apparatus for fractured Thigh . . . . . 441
Gieze, Counsellor, of *Dorpat*, on a new Metal extracted from the residue of Sulphuric Acid . . . . . . . . . 434
Girdlestone, Dr. of *Yarmouth*, case of Hydrocephalus Externus cured by Bandage . . . . . . . . . . 183
Godfrey, Mr. Surgeon of the *Cambrian*, case of a Man struck Deaf and Dumb by Lightning . . . . . . . 369
Goelis, Dr. of *Vienna*, Practical Dissertation on the principal Diseases of Children . . . . . . . . . . 77
Gomes, Dr. A. B. of *Lisbon*, Portugueze work on the means of checking Elephantiasis in Portugal . . . . . . . 516
Graaf, Dr. of *Cologne*, on Poisoning from Tincture of Cantharides . 437
Grafe, Prof. of *Berlin*, Removal of half of the lower Jaw from its Articulation . . . . . . . . . . 260
Granville, Dr. of *London*, on Sulphuretted Azote . . . . 189
Report of the Practice of Midwifery at the Westminster Dispensary 282, 374
Proposal for a new Classification of Medicines . . . . 289
On Encysted Tumors in the Ovarium, discharged through the Abdominal Parietes, in consequence of Suppuration . . . 463
Directions for conducting the Analysis of Vegetable Substances . 466
Greenhow, Mr. of *Newcastle*, case of Sanguineous Apoplexy in a Child . 181
Grotthus, Mr. on the Phosphorescence of the Lampyris Italica . . 261
Hall, Dr. Marshall, an Essay on the Symptoms and History of Diseases 401
Hamilton, Mr. on the Principles of Medicine . . . . . 219
Hare, Dr. of *Philadelphia*, on Heat produced on the Skin by Chlorine . 349
Hartman, Professor, Review of the Mind of Man . . . . 251
Henkesew, Dr. of *Hildesheim*, on the use of the Subnitrate of Bismuth in Agues . . . . . . . . . . . 436
Herbholt, Dr. of *Copenhagen*, on the use of Belladonna as a Preservative from Scarlet Fever . . . . . . . . . 336
Heuke, Dr. of *Erlangen*, a Manual towards the Knowledge and Treatment of the Diseases of Children, in German . . . . . 78
Heusman, Mr. of *Louvain*, on the Re-establishment of the Tincture of Iodine deteriorated by time, &c. . . . . . . 262
Home, Sir E. of *London*, Microscopical Observations on the Structure of the Eye 81
Account of a new Species of Rhinoceros, read to the Royal Society . ib.

PAGE
**Hood**, Dr. and Dr. **Sillar**, on the Decomposition of Aliment . . 184
**Howel**, Dr. of *Philadelphia*, on the Structure of the Capsule of the Lens 346
**Hufeland**, Dr. Address to the Members of the Medical Profession . 251
**Hutchison**, Mr. A. C. of *London*, description of Introsusceptio . 272
**Ivory**, J. Esq. Mathematical Paper read to the Royal Society . . 162
**Jenner**, Dr. **Robert**, on artificial Eruptions produced by Antimonial Ointment . . . . . . . . . . 244
**Jukes**, Mr. of *London*, case of Spina Bifida . . . . . 106
**Kergaradec**, Dr. of *Paris*, strong instance of the protecting Power of Vaccination against Small-pox . . . . . . . 432
L'Auscultation appliquée a l'Etude de la Grossesse . . 504
**Kinglake**, Dr. of *Taunton*, on maximum Doses in Medicine . . 89
On Hydrocephalic Affections . . . . . . 177
Remarks on Dr. Scudamore and Mr. Bampfield on Colchicum . 198
**Korlim**, Dr. of *Stalberg*, on Mare's Milk in Tænia . . . 523
**Kunze**, Dr. of *Prussia*, de Dysphagia Commentatio Pathologica . . 430
**Kuhn**, Prof. of *Leipsic*, Edition of the Works of Galen . . . 434
**Laennec**, Dr. of *Paris*, Translation of a Work on Diseases of the Chest 140
**Larrey**, Baron, of *Paris*, Memoirs of Surgery . . . . 76
**Leonhardhi**, Dr. of *Dresden*, Pharmacopœia Saxonica . . . 78
**Locock**, Dr. Dissertatio Medica inauguralis de Cordis Palpitatione . 70
**Macann**, Dr. Medicamina Officinalia . . . . . . 421
**Macartney**, Prof. of *Dublin*, mode of preserving Anatomical Preparations in a Solution of Alum and Nitre . . . . . . 436
**Macleod**, Dr. of *London*, Report of Diseases . . . . . 338
**Magendie**, Dr. of *Paris*, Journal of Physiology announced . . 335
**Mantovani**, Prof. Lectures on Inflammation . . . . . 151
**Mareçhal**, Mr. of *Montpellier*, Clinical Observations . . . 249
**Mazet**, Dr. of *Paris*, death of . . . . . . . . 166
**Meckel**, Professor, on Aberrations in the Distribution of the Blood-vessels 127
**Mecrieu**, Dr. of *Paris*, Memoir on the supposed Faculty of the Membranes of the Ovum in dilating the Os Uteri during Labour, read to the Atheneum of Medicine . . . . . . . . 432
**Mege**, M. of *Paris*, case of frontal Tic Douloureux, cured by the Sulphate of Quinine, reported to the Atheneum . . . . . ib.
**Miller**, Dr. C. of *Bristol*, on Phosphoric Acid in Jaundice . . 350
**Mondini**, Prof. on the Passage of the Bile into the Duodenum . . 252
**Mongellaz**, Dr. Essais sur les Irritations Intermittents . . . 414
**Morey**, Mr. of the *United States*, on artificial Mineral Waters . . 200
**Morrin**, M. of *Rouen*, Chemical Examination of the Humour of Porrigo 350
**Moscate**, M. of *Milan*, invention of an Instrument for Nasal Polypi . 84
**Most**, Dr. of *Hamburgh*, Treatise on Influenza Europœa . . . 158
**Murray**, Mr. J. Variation in the Thermometer at different Altitudes in the same Room . . . . . . . . . . 261
On the Solubility of Camphor in Sulphuret of Carbon . . 349
**Muscroft**, Mr. of *Pontecraft*, on Madness in the Human Subject, by the Absorption of the Matter of Glanders . . . . . 435
**Neumenn**, Dr. of *Berlin*, case of internal Abdominal Hemorrhage . 438
**Niel**, M. of *Montpellier*, on the Preparations of Gold in various Diseases 248
**Nimmo**, Dr. analysis of Croton Oil, and on its Purgative Properties . 378
**Norman**, Mr. on the pernicious Effects of Colchicum . . . . 459
**Orr**, Dr. **James**, of *Edinburgh*, on the use of Cubebs in Leucorrhœa . 434
**Palin**, Dr. Observations on the Influence of Habits and Manners, national and domestic, upon the Human Race . . . . . 315
**Parent**, **Duchatelet**, and **Martinez**, Drs. on Inflammation of the Spinal and Cerebral Arachnoid Membranes . . . . 495
**Patissier**, Dr. case of Sudden Death . . . . . . . 432
**Pedralbes**, Dr. of *Madrid*, on the State of the Medical Profession in Spain 80
**Person**, Dr. on the preservative Powers of the Scarabæus Melalontha against Hydrophobia . . . . . . . . 256
**Philip**, Dr. **Wilson**, on his Physiological Theories . . . 388
**Phillips**, Sir **Richard**, of *London*, Essays on the Proximate Causes of the Material Phenomena of the Universe . . . . . . 72

PAGE
PHILIPS and FARRADY, on a new Combination of Chlorine and Carbon . 80
PLAYER, Mr. on Irritation of the Spinal Nerves . . . . 301
PLUMBE, Mr. of *London*, a Practical Treatise on Porrigo, or Scald Head 146
Fatal case of Parturition . . . . . . . 276
Case of supposed Apoplexy . . . . . . . 367
POMMEN, Dr. of *Prussia*, attempt to improve the knowledge of Sporadic Typhus, &c. . . . . . . . . . 515
PORTER, Dr. of *Hunterdon county*, on the use of Oil of Turpentine as an internal Remedy in Psora . . . . . . . 83
PORTER, Dr. of *Carolina*, Tests for Arsenic . . . . . 260
PREVOST, Dr. J. of *Geneva*, on Spermatic Animalculi . . . 172
PRICE, Dr. REES, of *London*, Epitome of Pharmaceutical Chemistry . 334
PRING, Mr. of *Bath*, Result of an Operation for the making of an artificial Anus . . . . . . . . . . 109
PRITCHARD, Dr. on Diseases of the Nervous System . . . 129
PROCHASCA, Prof. Physiology of Human Nature . . . . 429
REIDMULLER, Dr. of *Nuremberg*, on the use of Black Pepper in Intermittent Fevers . . . . . . . . . 431
RENARD, Mr. of *Romans*, successful Operation of Gastrotomy . . 167
RENZY, Captain, Enchiridion, or a Hand for the one-Handed . . 69
REVELY, Mr. on the use of Soap in the Setting of Surgical Instruments . 434
RIGBY, Dr. of *Norwich*, Biographical Account of . . . . 163
RING, Mr. of *London*, Biographical Account of . . . . . 165
RISTELHUEBER, Dr. of *Strasbourg*, Rapports et Consultations de Medicine Legale . . . . . . . . . . 75
ROBINSON, Mr. of *Derby*, a beautiful Specimen of the great-eared Owl . 85
ROGERS, Mr. on Worms in the Pus of Pulmonary Consumption . . 176
RUDTORFFER, Prof. of *Vienna*, Armamentarium Chirurgicum Selectum . 78
SABINE, Mr. JOSEPH, Description of Quadrupeds and Birds sent from the Polar Regions . . . . . . . . . 171
SAVENKO, Dr. of *St. Petersburg*, Description of Surgical Instruments found in Pompeii . . . . . . . . . 353
SCARPA, Prof. of *Pavia*, sull' Ernio del Perineo . . . . . 79
SCHROEDER, Dr. of *Groningen*, on the Coagulation of the Blood . . . 159
SEMENTINI, Cavalier, on Nitrate of Silver in Epilepsy . . . . 524
SHIPMAN, Mr. of *London*, on Disorders of the Stomach . . . 246
SILLAR and HOOD, Drs. Experiments on the Decomposition of Aliment . 190
SMITH, Dr. NATHAN, of the *United States*, on the cure of Dropsy of the Ovarium by Extirpation of the Sac . . . . . 280
SOUBERVILLE, Dr. of *Paris*, on a singular Case of Lithotomy . . 300
SQUIRES, Mr. R. on a new Mineral Substance . . . . . 434
STEARNS, Dr. of the *United States*, on the Functions and Diseases of the Liver 123
STEPHENSON, Mr. of *Alcester*, on Introsusception . . . . . 274
SYDER, Mr. MINGAY, a Series of Questions and Answers for Students . 74
A Series of Lectures on Surgery . . . . . . 228
TADDEI, Dr. Preparation of Ethiop's Mineral . . . . 435
TELFORD, Mr. on the Mal d'Estomac . . . . . . 450
THOMSON, Mr. A. T. London Dispensatory . . . . . 510
THOMSON, Dr. of *Glasgow*, Answer to a Review of Mr. Brande . . 511
THOMPSON, Mr. HENRY, of *Enfield*, Nursery Guide . . . 508
TROTTER, Dr. of *Newcastle*, on Diabetes . . . . . . 460
TWEDDALE, Mr. case of Paralysis of the Kidneys . . . 107
TYTLER, Dr. of *India*, Exemplum Parvulum Nosologiæ . . 265
VANDERZADE, Dr. of *Antwerp*, on Puerperal Fever . . . . 517
VIREY, Prof. of *Paris*, History of the Manners and Instincts of Animals 158
WEBER, Prof. of *Leipsic*, on the Organs of Hearing . . . . 438
WEINHOLD, Dr. of *Halle*, Eighth Report of the most remarkable Cases in the Clinical Institution . . . . . . . . 159
WHITE, Mr. of *Bath*, Observations on Stricture of the Rectum . . 324
WILKINSON, Mr. of *London*, on Cutaneous Diseases . . . 392
YEATMAN, Mr. of *Frome*, on Sutures in lacerated Wounds . . 447
WILLAN, Dr. (the late,) of *London*, Miscellaneous Works . . . 62
YELIN, Chevalier, of *Bavaria*, on the Magnetism of Needles by Electricity 85

### MONTHLY CATALOGUE OF MEDICAL BOOKS.

**A Treatise on Parturition; with three Engravings. 4to. 5s.**

**An Inquiry into the Opinions, Ancient and Modern, called Life and Organization. By John Barclay, M.D. 8vo. 14s.**

**A Treatise on the Diseases of the Chest; translated from the French of R. T. H. Laennec, M.D. With a Preface and Notes. By John Forbes, M.D. 8vo. 14s.**

**Remarks upon Morbus Oryzeus; or, Disease occasioned by the Employment of Noxious Rice as Food. By Robert Tytler, M.D. M.A.S. In two Parts. 8vo. 8s.**

**A Treatise on Diseases of the Nervous System. Comprising Convulsive and Maniacal Affections. By J. C. Prichard, M.D. Part I. 8vo. 12s.**

---

### METEOROLOGICAL JOURNAL.

**By Messrs. William Harris and Co. 50, Holborn, London.**

***From November* 20 *to December* 19, *inclusive.***

| Day of Month. | Moon. | Rain gauge | THERM. | | | BAROM. | | DeLuc's Hygro. | | WIND. | | ATMOSPHERIC VARIATION. | | |
|---|---|---|---|---|---|---|---|---|---|---|---|---|---|---|
| Nov | | | | | | | | | | | | | | |
| 20 | | | 50 | 52 | 52 | 29.97 | 29.78 | 54 | 54 | WSW | WSW | Fine | Cloud. | |
| 21 | | | 50 | 48 | 42 | 29.65 | 29.88 | 58 | 56 | NW | NW | Rain | Fine | Cloud |
| 22 | | | 46 | 54 | 46 | 29.55 | 29.61 | 57 | 56 | SW | W | Rain | Fine | |
| 23 | | .20 | 52 | 49 | 39 | 29.71 | 29.95 | 56 | 57 | W | NNW | Cloudy | Rain | Fine |
| 24 | | .25 | 46 | 50 | 43 | 29.72 | 29.74 | 57 | 56 | WSW | WNW | Rain | Cloud. | Fine |
| 25 | ● | .10 | 45 | 55 | 45 | 29.63 | 29.57 | 56 | 60 | NW | SW | Fine | | Rain |
| 26 | | | 53 | 55 | 49 | 29.38 | 29.26 | 62 | 60 | WSW | WSW | Cloudy | Rain | Cloud. |
| 27 | | | 48 | 50 | 36 | 29.51 | 29.90 | 56 | 55 | WNW | W | Cloudy | Sho'ry | Fine |
| 28 | | .27 | 43 | 50 | 49 | 29.81 | 29.74 | 55 | 60 | WSW | WSW | Cloudy | Rain | Fine |
| 29 | | | 52 | 52 | 48 | 29.69 | 29.61 | 65 | 60 | SW va. | SW va. | Cloudy | | Fine |
| 30 | | .09 | 50 | 52 | 49 | 29.55 | 29.47 | 55 | 57 | W var. | W | Cloudy | | Rain |
| Dec | | | | | | | | | | | | | | |
| 1 | | .20 | 52 | 53 | 41 | 29.63 | 29.81 | 53 | 55 | NW va. | W | Fine | Rain | |
| 2 | ☾ | | 43 | 49 | 39 | 29.90 | 30.02 | 54 | 53 | W var. | W | Cloudy | Fine | |
| 3 | | .16 | 41 | 48 | 36 | 29.88 | 29.80 | 55 | 55 | W var. | WNW | Cloudy | Rain | Fine |
| 4 | | .26 | 40 | 48 | 37 | 29.82 | 29.76 | 56 | 60 | SW | W | Fine | | Rain |
| 5 | | .41 | 41 | 48 | 36 | 29.87 | 29.95 | 55 | 55 | W | NW | Showery | R.&Li. | Fine |
| 6 | | | 39 | 42 | 40 | 30.20 | 30.30 | 53 | 55 | NE | SE | Fine | | |
| 7 | | .10 | 40 | 49 | 44 | 30.19 | 30.11 | 57 | 55 | S | SSW | Showery | | Cloud. |
| 8 | | | 43 | 53 | 49 | 30.04 | 30.12 | 62 | 63 | W | W | Cloudy | | |
| 9 | ○ | | 50 | 51 | 41 | 30.10 | 30.07 | 65 | 65 | SW | SW | Showery | | |
| 10 | | .08 | 42 | 46 | 38 | 30.00 | 29.95 | 61 | 61 | W | W | Fine | | Sho'ry |
| 11 | | | 39 | 48 | 40 | 30.21 | 30.35 | 60 | 56 | S | SSE | Cloudy | Fine | Cloud. |
| 12 | | | 43 | 52 | 41 | 30.20 | 30.09 | 61 | 63 | S | S | Cloudy | Fine | Cloud. |
| 13 | | .06 | 42 | 51 | 43 | 30.00 | 30.02 | 62 | 63 | SE | SE | Cloudy | | Sho'ry |
| 14 | | .16 | 43 | 50 | 46 | 30.03 | 29.97 | 61 | 61 | S | SSW | Cloudy | Rain | Cloud. |
| 15 | | | 47 | 51 | 46 | 29.99 | 29.85 | 63 | 60 | S | S | Cloudy | | |
| 16 | ☽ | | 48 | 52 | 49 | 29.71 | 29.63 | 61 | 58 | W | SW | Very fine | | Cloud. |
| 17 | | .11 | 50 | 53 | 49 | 29.52 | 29.32 | 63 | 62 | SW | SW | Rain | | Cloud. |
| 18 | | .18 | 51 | 52 | 45 | 29.25 | 29.13 | 61 | 57 | SW va. | SW | Rain | | |
| 19 | | .13 | 46 | 49 | 42 | 29.21 | 29.33 | 63 | 65 | SW | W | Rain | Sho'ry | |

The quantity of rain fallen in the month of November, was 3 inches and 59-100ths.

### NOTICES TO CORRESPONDENTS.

*A letter has been handed to us by our Publisher, signed* I. C. H., *in which the writer, in terms as gentle as they are grammatical, abuses* SHAKSPEARE, Dr. BRADLEY, *the friends and the enemies of vaccination,* Dr. JAMES JOHNSON, Dr. W. HUTCHINSON, *and poor unoffending ourselves. In this specimen of epistolary jumble, in which the only really remarkable things are* two *or* three *expressions of* correct English, "pauci natantes in gurgite vasto,"—*we have not been able to discover the real motives of* I. C. H.'s *lamentation, unless it be* Othello's *imprecation against empirics, quoted in our last* Notices to Correspondents. Mr. H. *says, that he is a respectable surgeon of twenty years' standing, intending to* retire *with an honorary diploma; and he cunningly adds, " and I should be glad to know where I can get one for nothing."—Certain diplomas are already too great a bargain; and neither he, nor any of his fellow respectable practitioners, who are anxious to be styled* doctors, *have reason to complain.*

*Our best thanks are due to* Dr. KINGLAKE, Mr. PRING, *and others, for their valuable communications. The arrangements made for the present Number will serve as an apology for not inserting original papers, many of which will be published in the Number for February.* Mr. PRING *may rest assured, that we shall see his m's, n's, and w's, righted in their proper number of legs.*

Mr. BAMPFIELD's *observations on* Dr. SCUDAMORE's *reply to his remarks on Colchicum in our next.*

Dr. REEDER *has done us the honour of writing to us on the subject of our critical remarks on his book on Diseases of the Heart; and informs us, that " both the work and preface were composed entirely by himself." We never said that they were* not. *We only expressed our conviction, that the man who could write* such *a preface could not compose so respectable a work.* Dr. REEDER's *assertion, of course, proves that we were mistaken. But he has neither defended* his *preface, nor accounted for the singular omission of* LAENNEC's *name throughout his work!*

*We feel much obliged to* Mr. CHRISTIAN, *of Liverpool, for his communication, of which we have availed ourselves in our present Number. We can only say, that we entertain the most unfeigned respect for himself and his worthy colleagues at the Liverpool Ophthalmic Infirmary; and that we will be ever ready to pay attention to their suggestions, and shall be glad to hear from them on any future occasion.*

*In reply to the gentleman who wrote to us from the Literary and Philosophical Society of Newcastle, on the subject of our paper on the opening of an Egyptian Mummy, we beg to inform him, and the members of that learned body, that, at the time the paper in question was drawn up, no dissection of the mummy itself had been made; and that, since the dissection has taken place, so many have been the curious facts that have come to light, in consequence of it,—such the afflux of scientific and medical persons to see its results,—and so various the experiments that it has, of necessity, given rise to,—that the Editor has not been able to offer a continuation of his account to the readers of the London Medical and Physical Journal, as he originally intended. Should his occupations, however, allow it, the Editor hopes to have ready a full account of the whole proceedings, to be laid before the Royal Society, early in January. For the flattering expressions contained in* Mr. GREENHOW's *letter, we are most thankful.*

Dr. POWER, *of Queen-square, Westminster, has sent us a very angry reply to the review of his book contained in this Journal for the month of September. We have returned it as inadmissible, for a hundred reasons; three of which, only, we will enumerate. In the first place, the gentleman who published the review has resigned his office as Editor of the Journal, and is no longer in England; and it would be hard, indeed, if the present Editor were to be accountable for what he declares, most solemnly, to have had no hand in. In the second place, the reply is too much loaded with coarse invectives and personal allusions, which have even the demerit of being* manquées, *as the reviewer is* not *a practitioner in midwifery, and, consequently, could experience no feelings of envy at* Dr. POWER's *success. And, in the third place, there is such a disregard of grammar, syntax, and sense, in the reply, that, indifferently as we ourselves profess to write, it could not have failed to reflect on our judgment, had we given it insertion. In the second page only, we twice met with "you* was *pleased;"—with " independent" for " independently;" and with " the inflicting of an injury on Dr. Power's views!" Besides,* Dr. POWER *has not said a single word to contradict many of the strong assertions contained in the review in question; and, instead of complaining of its severity, (which, by the bye, he seems to have overlooked in a contemporary Journal,) he ought to be thankful that things are not worse. Is* Dr. POWER *serious in saying, though not in so many words, that menstruation is the weeping of the womb disappointed of impregnation!*—Proh! Pudor!

